# Improved oral health in head and neck cancer survivors (OHHNC study): a study protocol for a multicentre two-armed randomised controlled trial

**DOI:** 10.1186/s12885-026-16865-8

**Published:** 2026-09-07

**Authors:** Sandra Einarsson, Annica Almståhl, Hanna Gyllensten, Mats Sjöström, Lena Mårell, Ove Björ, Linda Marklund, Lovisa Farnebo, Göran Laurell, Ylva Tiblom Ehrsson

**Affiliations:** 1 Department of Food, Nutrition and Culinary Science, Umeå University, Umeå, Sweden; 2 Department of Oral Microbiology and Immunology, Institute of Odontology, Sahlgrenska Academy, University of Gothenburg, Göteborg, Sweden; 3 Department of Oral Health, Faculty of Odontology, Malmö University, Malmö, Sweden; 4 Institute of Health and Care Sciences Sahlgrenska Academy, University of Gothenburg, Göteborg, Sweden; 5 Department of Odontology, Oral and Maxillofacial surgery, Umeå University, Umeå, Sweden; 6 Department of Odontology, Orofacial Medicine, Umeå University, Umeå, Sweden; 7 Department of Diagnostics and Interventions/Oncology, Umeå University, Umeå, Sweden; 8 Department of Surgical Sciences, Section of Otorhinolaryngology and Head and Neck Surgery, Uppsala University, Uppsala, Sweden; 9 Department of Clinical Sciences, Division of ENT Diseases, Intervention and Technology, Karolinska Institutet, Stockholm, Sweden; 10 Medical Unit Head Neck, Lung and Skin Cancer, Karolinska University Hospital, Stockholm, Sweden; 11 Department of Biomedical and Clinical Sciences, Faculty of Medicine and Health Sciences, Division of Sensory Organs and Communication, Linköping University, Linköping, Sweden; 12 Department of Otorhinolaryngology, Region Östergötland Anesthetics, Operations, and Specialty Surgery Center, Linköping, Sweden

**Keywords:** Head and neck cancer, Oral health, Patient-reported outcome measures, Rehabilitation

## Abstract

**Background:**

Head and neck cancer comprises a heterogeneous group of tumours affecting anatomical sites of the upper aerodigestive tract. Treatment often involves surgery and either radiotherapy or chemoradiotherapy. These treatments are associated with substantial morbidity and may have long-lasting adverse effects on oral health. Oral complications can negatively affect health-related quality of life, nutritional status, and social and work-related functioning. The aim is to evaluate an oral health care programme designed to prevent or mitigate late oral sequalae, and to assess its impact on health-related quality of life, oral health, work ability, nutritional status, healthcare-related economic costs, and psychological well-being.

**Methods:**

This is a study protocol for a Swedish multicentre, two-armed, superiority randomised controlled trial that has been ethically approved by the Swedish Ethical Review Authority. Patient inclusion commenced in September 2025, with an intended sample of 300 participants. Adult patients (≥ 18 years) planned for curative-intent treatment for head and neck cancer (oral cavity, oropharynx, larynx stage III and IV, hypopharynx, nasopharynx, salivary gland, or unknown primary) are randomised (1:1) to either an oral health care programme (intervention) or standard of care. Exclusion criteria: severe alcoholism, cognitive impairment, or inability to understand the Swedish language. Participants are followed before the start of cancer treatment and at 4 follow-ups: 6 months after completion of treatment, and 1, 2, and 3 years after the 6-month follow-up. The primary outcome measure is health-related quality of life. Secondary outcomes include quantitative oral health indicators, return to work, nutritional status, healthcare-related economic costs, and patient-reported outcome measures focusing on self-reported oral health and psychological well-being (depression, anxiety and stress), and self-efficacy.

**Discussion:**

Oral health follow-up for head and neck cancer survivors often lacks consistency and uniformity. To date, few oral health care interventions for head and neck cancer survivors have been evaluated within the framework of a randomised controlled trial. The findings have the potential to inform clinical practice and contribute to the development of more integrated, evidence-based oral healthcare strategies within routine follow-up for head and neck cancer survivors, with the overarching goal of improving their oral health and long-term quality of life.

**Trial registration:**

ClinicalTrials.gov NCT07173270, date 15/9/2025.

**Graphical Abstract:**

**Figure:**
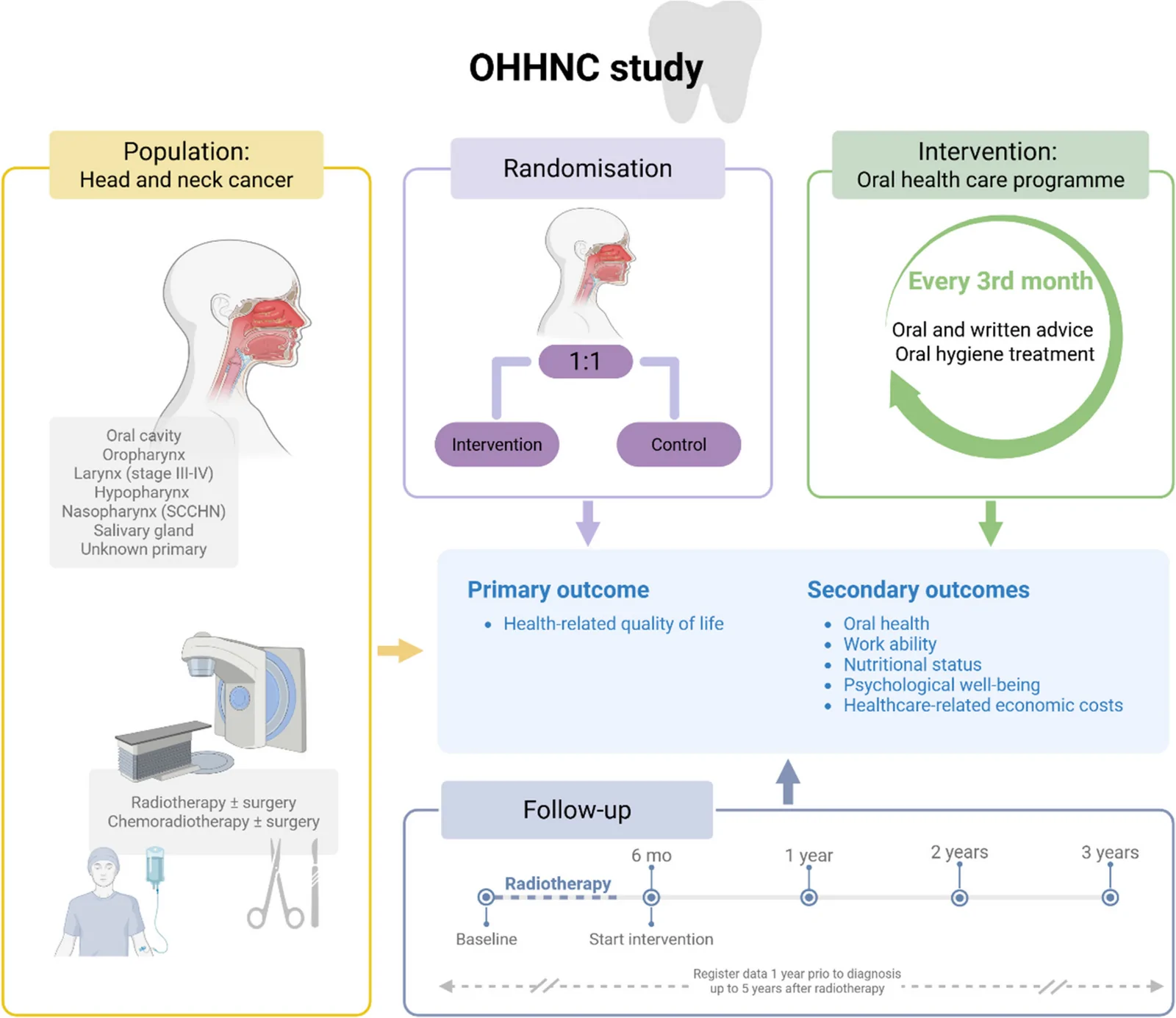
Created in BioRender. Tiblom Ehrsson, Y. (2026) https://BioRender.com/q3ajqtm

**Supplementary Information:**

The online version contains supplementary material available at https://doi.org/10.1186/s12885-026-16865-8.

## Background

Head and neck cancer (HNC) consists of a diverse group of tumours affecting anatomical sites of the upper aerodigestive tract, including the oral cavity, pharynx, larynx, nasal cavity, paranasal sinuses, and salivary glands [1, 2]. Treatment of HNC may be given as a single modality, but often requires a multimodal approach using surgery and adjuvant radiotherapy (RT) or chemoradiotherapy (CRT) [2]. The ultimate goal is to achieve cancer cure while keeping treatment-related morbidity to a minimum. However, given the many vital functions of the upper aerodigestive tract, treatment of HNC subsequently impacts patients’ functional capacity, well-being, and quality of life. Although treatment-related morbidity in HNC is complex and diverse, our particular focus here is on the challenges and symptoms related to the oral cavity, and more specifically, in relation to oral health status.

Despite efforts being made to deliver the prescribed irradiation dose volume to the tumour while minimising exposure to surrounding normal tissue, complete avoidance of irradiation to healthy tissue is not possible, which consequently leads to both acute and late treatment sequelae. Oral mucositis is a common acute treatment side effect of RT that subsequently progresses over the course of RT from mild (oral soreness, erythema) to severe (ulcerations) [3, 4]. The peak of oral mucositis is often observed near the end of RT treatment and usually resolves within a couple of months after completion [5]. Further, irradiation of the salivary glands may lead to hyposalivation [3] and possible permanent salivary gland hypofunction [6]. RT may also induce significant changes in the oral microbiota, with increased colonisation of pathogenic microorganisms and subsequent decline in oral health-related bacteria, which may exacerbate oral complications [7].

Late sequelae of RT include, for example, fibrosis of the masticatory muscles, which can result in trismus [4]. Trismus indicates severely restricted mouth opening, i.e., a mouth opening of 35 mm or less [8] and often progresses during the course of RT and for up to 6 months after start [9]. Osteoradionecrosis, resulting from irradiation of the jawbone, is another severe long-term complication from RT [4], often triggered by periodontal disease and dental extractions [10]. Irradiation of the teeth may also cause damage to hard dental tissue [11].

Together, acute and late sequelae from RT severely impact patients’ ability to sustain dental and periodontal integrity and make those treated for HNC vulnerable to poor oral health. For example, oral mucositis and trismus can impair the patient’s ability to perform oral hygiene and deliver dental care [4, 12]. Radiation-induced damage to dental hard tissues significantly increases the risk of dental caries [4], with an increase in the prevalence of caries over time [13]. Hyposalivation also contributes to poor oral health, as saliva is needed to preserve dental hard tissue integrity, protect the oral tissues, cleanse the oral cavity, and remove food particles [6]. Hence, the multitude of challenges and symptoms arising from HNC treatment that affect the oral cavity may exert a profound influence on the physical functioning and psychosocial well-being of HNC survivors, ultimately compromising overall quality of life.

Many HNC survivors testify that poor oral health is a major and chronic problem that affects many significant aspects of life due to a dry, foul-smelling oral cavity, pain, and missing teeth. Patients also report feeling insufficiently informed about the long-term effects of RT on their oral and dental health [14]. Physical dysfunction, e.g., pain due to mucositis and difficulty swallowing, may increase the risk of disease-related malnutrition [15, 16]. Social aspects can also be affected as some HNC survivors refrain from attending social events and from eating together with others when experiencing problems related to the oral cavity [17]. The numerous oral problems experienced by HNC survivors significantly affect health-related quality of life (HRQoL) [18, 19].

In accordance with standard clinical practice in Sweden, all patients with HNC scheduled to receive RT are routinely invited to undergo an oral health assessment prior to initiation of cancer treatment. Teeth with extensive periapical disease, moderate to severe attachment loss, or severe decay are extracted, and manifest caries lesions are treated. Conventional periodontal therapy is administered if necessary. Standard of care often includes follow-up at a specialist dental clinic for up to approximately three months after treatment. After this, patients are generally advised to visit their own dental clinic, when dental care costs are typically paid for by the patients themselves. Consequently, oral health follow-up for HNC survivors in Sweden lacks consistency and uniformity, and its frequency is variable across healthcare regions. Many patients are unable to afford dental care or choose not to prioritise it. The lack of support and the financial burden associated with dental care for patients with HNC are increasingly recognised within the research community [20, 21]. To support oral health and facilitate early detection of dental caries, patients with HNC should receive a comprehensive oral health care plan with frequent follow-ups over a long period of time after the completion of cancer treatment.

Well-defined, evidence-based guidelines across the healthcare system, including dental services, are essential to support HNC survivors in managing complex oral health challenges. However, there is currently limited evidence regarding how to implement such strategies effectively. This is, therefore, a protocol for a multicentre two-armed randomised controlled trial (RCT) with the aim of evaluating an oral health care programme designed to prevent or mitigate late oral sequalae following completion of HNC treatment, and assessing its impact on HRQoL, oral health, work ability, nutritional status, psychological well-being, and healthcare-related economic costs. Conducting a RCT to test this programme will provide valuable evidence for how structured, evidence-based interventions can be integrated into survivorship care to preserve oral health and improve long-term outcomes. We hypothesise that a clinically structured programme for HNC survivors will significantly improve both oral health and HRQoL.

## Methods

### Study design

We are conducting a multicentre two-armed superiority RCT (ClinicalTrials.gov NCT07173270) and this study protocol outlines the methods that will be used to evaluate the effectiveness of an oral health care programme designed to prevent or reduce late oral sequelae in HNC survivors. The research project has been approved by the Swedish Ethical Review Authority (Dnr 2023-05510-01), with an approved amendment (Dnr 2025-03485-02), and follows the Helsinki Declaration [22]. Written and oral information about the study is given to eligible participants, and written consent is mandatory for participation. This protocol follows the SPIRIT (Standard Protocol Items: Recommendations for Interventional Trials) guideline [23], and the SPIRIT checklist can be found as a supplement (Supplementary A).

### Participants

Patients diagnosed with HNC are randomly assigned in a 1:1 ratio to either an oral health care programme (intervention) or standard of care. The power analysis was performed based on data from a prospective observational study on HNC (ClinicalTrials.gov NCT03343236) involving individuals with various HNC diagnoses. In that study, we collected data using the European Organisation for Research and Treatment of Cancer Quality of Life Questionnaire – Head and Neck Module (EORTC QLQ-H&N35) [24]. Data from this instrument, specifically the ‘Dry mouth component’ of the EORTC QLQ-H&N35, form the basis for the power calculation, assuming a mean of 52 and a standard deviation (SD) of 33 after one year in the standard of care group.

Sample size was determined using simulation-based power calculations for an ANCOVA model, adjusting for baseline values, with robust standard errors. Simulations enable a skewed outcome bounded between 0 and 100 and a conservative zero correlation between baseline and follow-up. Simulation-based power calculations were chosen due to the discrete and bounded nature of the outcome variable, which violates the assumptions underlying standard normal-theory sample size formulas.

A total of 250 participants (125 per group) provides approximately 80% power to detect a clinically relevant difference of 10 units between groups at a two-sided 5% significance level. To account for potential dropouts, we plan to enrol 300 patients in total that will be randomised. Calculations were performed using R version 4.3.3.

Inclusion started in September 2025. Patients are recruited at different hospitals in Sweden. Clinical/research nurses at each cite identify eligible patients in relation to a multidisciplinary conference, where treatment options for newly diagnosed patients with HNC are discussed and planned. Informed consent is obtained from the participant by the clinical/research nurses. The number of patients who decline participation or who are inadvertently omitted from being approached is documented in the research database Research Electronic Data Capture (REDCap) [25].

#### Inclusion and exclusion criteria

The inclusion criteria are patients (≥ 18 years) planned for HNC treatment with curative intent, i.e., RT with or without systemic anti-cancer therapy, SACT (e.g., chemotherapy, immune therapy, targeted therapy) and/or surgery. Tumour sites include the oral cavity, oropharynx, larynx (only stage III and IV), hypopharynx, nasopharynx (squamous cell carcinoma), salivary glands, or unknown primary. The exclusion criteria are patients with severe alcoholism, cognitive impairment, or inability to understand the Swedish language.

### Allocation and follow-up

**Fig. 1:**
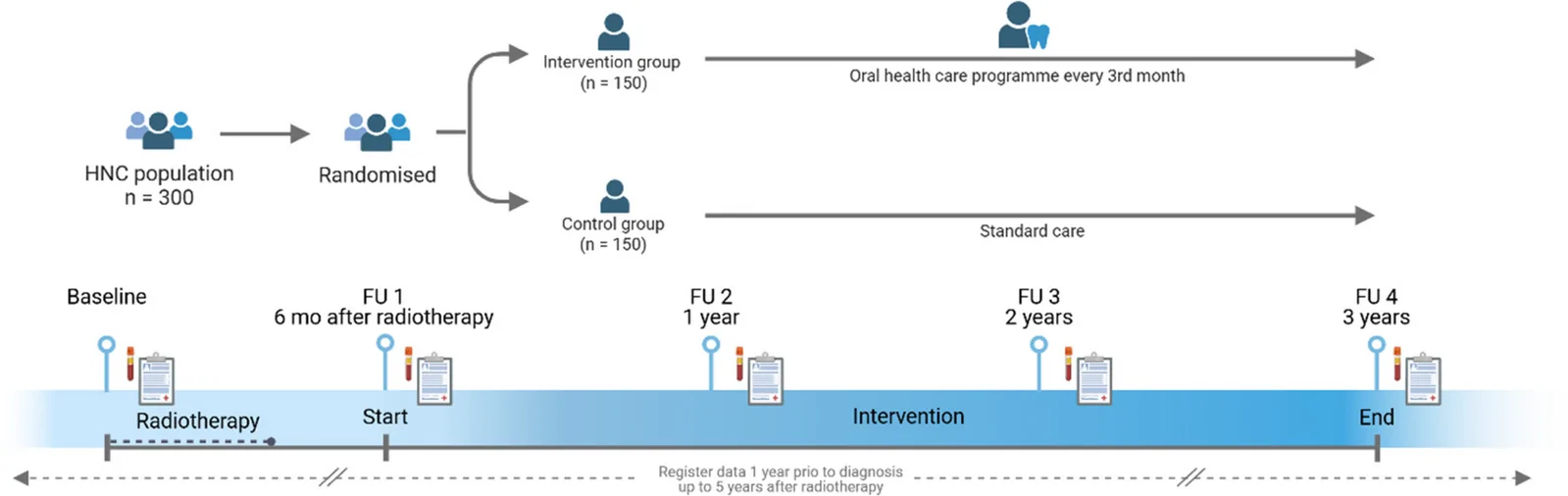
Follow-up and randomisation to the intervention; an oral health care programme. Created in BioRender. Tiblom Ehrsson, Y. (2026) https://BioRender.com/v8xvrby

Patients are followed from baseline (before the start of cancer treatment) and at 4 subsequent follow-ups: 6 months after completion of treatment, and 1, 2, and 3 years after the 6-month follow-up (Fig. 1).

Standard of care, according to clinical practice in Sweden, is provided to all participants for up to approximately 3 months after treatment. At the 6-month follow-up, patients are randomised (1:1) to receive either the intervention (oral health care programme) or standard of care. A statistician has generated the randomisation list; no members of the research team were involved in this process or have access to the randomisation list. Randomisation is computer-generated using REDCap.

### Intervention and standard of care

The oral health care programme is a practice-led intervention that was developed by a multidisciplinary team of healthcare professionals and researchers (physicians, nurses, dentists, dental hygienists, and a dietitian), and in close collaboration with HNC survivors. The intervention starts 6 months after completion of RT and ends after the 3-year follow-up. Every 3 months, participants in the intervention group are scheduled for an appointment with a dental hygienist. During these visits, participants are asked about oral hygiene habits, fluoride use, dietary habits (with focus on intake of easily degradable carbohydrates), and oral dryness. The dental hygienist also performs a thorough examination of the oral cavity, assessing signs of dental problems (e.g., tooth decay) or gum problems (e.g., gingivitis or periodontitis).

The intervention is individually tailored and based on the examination and the patient’s report. It includes oral and written advice, and recommendations from the dental hygienist, e.g., how to increase oral hygiene using the correct toothbrushing technique, interdental cleaning, or strategies to address oral dryness. It also includes tailored preventive professional oral hygiene treatment, e.g., plaque and dental calculus removal, fluoride gel application, and polishing to produce a smooth and clean surface. All visits to the dental hygienist are at no cost. If oral problems are beyond the competence of the dental hygienist, dentists are consulted and the participant pays for any necessary treatment.

At the 6-month follow-up, patients are randomised to receive the intervention (oral health care programme) or standard of care, which in Sweden only includes dental care for up to approximately 3 months following treatment. Within standard of care, patients therefore need to actively seek dental care and support themselves after 6 months. Patients who have undergone RT targeting the oral cavity may be entitled to a partial allowance, which subsidies preventive dental care; however, not all patients receive this benefit, and it does not cover all the costs.

### Data collection

#### Primary and secondary outcome measures

The primary outcome measure is HRQoL. Secondary outcome measures are: Quantitative oral health indicators, return to work (sick leave), nutritional status, healthcare-related economic costs, and patient-reported outcome measures (PROMs) focusing on self-reported oral health, psychological well-being (depression, anxiety and stress), and self-efficacy.

#### Data collection overview

Clinical/research nurses, dentists and dental hygienists at each site are responsible for the data collection. Data are collected from all participants at baseline, 6 months after the end of treatment (randomisation and start of intervention), and 1, 2 and 3 years after randomisation. Non-adherence and non-retention are registered.

Data gathered through history taking or medical records include, e.g., demographics (sex, age and education), as well as information on diagnosis and treatment (tumour site and stage, treatment modality, comorbidities and medication), work ability and sick leave, eating ability, and habits, including use of nicotine and alcohol, nutritional status (anthropometrics, body composition). Body composition measurements using calf circumference are performed on all participants. Bioelectrical impedance analysis (Bodystat^®^ Multiscan 5000) is used on a subgroup.

In addition, participants complete questionnaires (PROMs) to assess HRQoL, depression, anxiety and stress, health status, self-efficacy, and self-rated oral health (Table 1). The questionnaires are completed digitally or on paper.

**Table 1:** Data overview from patient-reported outcome measures, PROMs

Questionnaire	Description
European Organization for Research and Treatment of Cancer Quality of Life Questionnaire-Core 30 (EORTC-C30) [26]	Assesses health-related quality of life in cancer patients. Comprises 30 items covering, e.g., global health status, functional domains (physical, role, emotional, cognitive, and social), symptom scales (fatigue, pain, nausea/vomiting).
EORTC-Head and Neck Cancer Module (EORTC-HN43) [27]	Assesses health-related quality of life in patients with head and neck cancer, an updated version of HN35. Includes 43 items addressing disease- and treatment-specific issues, such as swallowing, speech, senses (taste and smell), pain, social eating, sexuality, and body image, as well as symptoms such as dry mouth and sticky saliva.
Depression Anxiety Stress Scale-21 (DASS-21) [28]	Measures negative emotional states. Consists of 21 items divided into three subscales assessing symptoms of depression, anxiety, and stress.
EQ-5D-3 L [29, 30]	Measures general health status. Consists of five dimensions (mobility, self-care, usual activities, pain/discomfort, and anxiety/depression). Each dimension has three levels of severity.
General Self-Efficacy (GSE) [31]	Assesses an individual’s perceived ability to cope with a variety of demanding situations. Consists of 10 items measuring self-belief related to problem-solving and adaptation.
Self-Perceived Oral Health (SPOH) [32]	A modified version of the SPOH instrument is used to assess subjective oral health. The instrument includes items on self-rated oral health (12 items), oral health behaviours (8 items), perceived oral health knowledge (4 items), and future perspectives (2 items).

Data on oral health (number of remaining teeth, maximum interincisal opening [MIO], caries and periodontal status) are collected during clinical examination. Participants are asked how often they have sought dental care. Computed Tomography (CT) and Cone-Beam Computed Tomography (CBCT) are used to detect pathological changes in the jaw (osteoradionecrosis). Blood and saliva samples are collected for analysis and long-term storage (biobanking). Blood samples are analysed for haematology and immune parameters, including markers of inflammation. The stimulated whole salivary secretion rate is determined, and the buffering capacity is assessed. For a subgroup of participants, saliva composition (proteins, ions, metabolites and inflammatory biomarkers) and the oral microbiome will be analysed.

Register data will be obtained from the National Board of Health and Welfare (National Prescribed Drug Register, National Register of Care and Social Services for the Elderly and Persons with Impairments, National Register of Interventions in Municipal Health Care, National Cause of Death Register, National Patient Register, National Dental Health Register) and from the Swedish Social Insurance Agency (Micro Data for Analysis of Social Insurance [MiDAS] database, Dental care). Register data on healthcare utilisation will also be obtained for a subgroup of patients. Register linkage will be performed using personal identity numbers, and the data will be pseudonymised after data collection.

The collected register data will cover the period from one year prior to diagnosis to five years after the completion of treatment, and will include information about, for example, prescribed drugs, municipal health and social care, specialised outpatient and inpatient care, registered sickness absence, and costs related to functional impairments, as well as dental healthcare.

##### Data security

Data are collected using a secure, web-based platform (REDCap) designed to create and manage research databases [25]. REDCap is also used for randomisation. The system supports validated data entry, audit trails, automated export to statistical software, and advanced features for longitudinal data collection and multi-site collaboration. Confidentiality is ensured by using coding lists with limited access. All data handling is compliant with the European Union General Data Protection Regulation (GDPR, 2016/679).

### Feasibility testing

The feasibility of the intervention will be tested after the 6-month follow-up, once participants have been randomised. The first randomised participants will be asked to take part in the feasibility testing. We will use individual and/or focus group interviews with a sample of participants (*n* = 10) and also with a subgroup of healthcare providers responsible for recruiting participants to the study and conducting the data collection and intervention (dental hygienists, clinical/research nurses, *n* = 10). Interviews will follow a semi-structured format based on a predefined guide. The aim is to explore the feasibility and acceptability of the data collection procedures for primary and secondary outcome measures, as well as the intervention itself [33]. Additionally, we will review recruitment and retention rates, completeness of outcome measure data, and practical aspects of data collection. Protocol modifications will be implemented if suggested by the feasibility testing results. Data from the HNC survivors participating in the feasibility testing will be included in the final analysis, provided that any adjustments to the oral health care programme do not compromise the study’s integrity.

### Data processing and analysis

The effects of the intervention will be evaluated in comparison with standard of care. Outcomes will be analysed using either change‑from‑baseline or end‑point values, depending on the nature of each variable. Time‑to‑event outcomes, where applicable, will use time from randomisation to the occurrence of the event of interest.

The main statistical analyses will be performed by statisticians according to the intention-to-treat (ITT) principle. The primary analysis of the EORTC QLQ-HN43 [27] will be conducted using an ANCOVA model adjusting for baseline values, with robust standard errors. Analyses will be performed at each follow-up time point, with the primary comparison at 12 months. To account for within-subject correlation, a linear mixed-effects model with random subject intercepts and a treatment-by-time interaction, using all three follow-up time points, will be used as a sensitivity analysis.

Missing data will be addressed using multiple imputation under the assumption that data are missing at random. Effect size will be estimated using Cohen’s d. Sensitivity analyses to assess the impact of non-compliance will include complete-case and per-protocol analyses. Descriptive statistics will be presented as numbers and percentages for categorical variables, and as means with standard deviation (SD) for continuous variables.

Resource use and societal costs will be used to investigate cost-effectiveness. The economic evaluation will be based on the direct medical costs of specialised healthcare (calculated from diagnosis-related group weights) and drug costs (the register includes costs for dispensed prescription drugs) among HNC survivors, as well as the direct non-medical costs of formal assistance services (assistance and home care, costed using templates from national and municipal statistics), costs for dental care, and indirect costs in the form of productivity loss for sickness absence, estimated according to the human capital approach. A subgroup analysis will be conducted using administrative data on primary care use from one healthcare region and unit costs for such use from national statistics, to identify the effect of including primary care in the evaluation. The economic evaluation will estimate societal costs over a one-year time horizon for each patient; no discounting will therefore be necessary in these analyses. Societal costs will be reported, as well as costs by components and payers.

The economic evaluation will compare differences in costs with differences in health, measured as Quality‑Adjusted Life Years (QALYs), which will be estimated using both the EORTC-30 [26] and the EQ-5D-3 L (Table 1) [29, 30]. Health status will be translated into QALYs using established methods and value sets. Cost-effectiveness will be analysed using methods that can manage skewed cost data and, where necessary, impute missing data. The outcome will be reported as an incremental cost-effectiveness ratio showing the cost of achieving a given health gain, a cost-effectiveness plane illustrating the cost and health components, and cost-effectiveness acceptability curves in cases such as improved health at increased cost. Stochastic and probabilistic sensitivity analyses will be performed to investigate the effects of assumptions made in the analysis, such as price levels for different resources used, the value set for converting health status to QALYs, and heterogeneity within the patient population.

Qualitative data from the feasibility testing will be transcribed verbatim and analysed using thematic analysis [34]. Quantitative data from the feasibility trial will be analysed descriptively to optimise any data collection procedures.

## Discussion

Advancements in HNC diagnostics and treatments have led to an improvement in survival over the past decade [35]. These improvements are highly creditable but need to be followed by effective aftercare of cancer survivors, since functional and financial vulnerability are strongly associated with survivorship. From previous research, it is evident that many HNC survivors experience long-term oral sequelae, such as hyposalivation, pain, and radiation-induced dental caries [4]. These complications significantly impact HRQoL, even after successful cancer treatment [18, 19]. Although substantial evidence underscores the critical role of dental care for HNC survivors, its systematic integration into oncologic treatment regimens remains a complex and persistent challenge.

To date, few interventions targeting oral health in HNC have been evaluated using RCT methodology [36–38]. All the identified studies included fewer than 100 participants, and none followed patients for more than one year. Reported interventions comprised either professional oral care (e.g., tooth brushing instructions, professional mechanical tooth cleaning, and fluoride varnish application) [37], an integrated supportive programme (including oral hygiene instructions, oral health self-care strategies, facial and tongue muscle exercises, and salivary gland massage) [36], or an oral health programme (with oral health education, fluoride varnish application, and fluoride mouth rinsing) [38]. To the best of our knowledge, this will be the first RCT among HNC survivors to evaluate a comprehensive oral health program and its impact on both physical and psychological well-being over an extended follow-up period. If the intervention demonstrates promising outcomes, it will provide novel insights into effective strategies to mitigate oral complications and improve long-term oral health and quality of life in this population.

There are important methodological considerations that need to be acknowledged. Given the study design and intervention, blinding will not be feasible. This will pose a particular challenge regarding the PROMs [39]. When an intervention is delivered through direct human interaction, there is an inherent risk that attention bias will affect PROMs. Hence, improvements in PROM scores may not solely reflect the intervention itself but could also result from increased frequency of patient–provider contact. Additionally, patients who are aware of receiving an intervention may report improvements more positively. When using PROMs, there is a risk that patients may adapt to their condition or treatment sequelae, leading to changes in PROM scores that reflect adaption rather than true changes in symptoms. We believe that using PROM instruments will add substantial value and enable us to demonstrate the therapeutic benefit of the oral health care programme from the patient’s perspective. To address the previously described potential biases associated with using PROMs, and to add objectivity, the outcomes measured are not based solely on PROMs but instead on a combination of patient-reported outcomes, clinical indicators, imaging, and biochemical biomarkers.

The extensive data collection is a strength and will enable us to investigate the longitudinal impact of the oral health care programme. To enhance the generalizability of the findings, a diverse and inclusive cohort of patients with HNC, representing different tumour sites, stages, and treatment modalities, will be recruited. We will use REDCap [25] for randomisation, which enables instant and consistent allocation according to a predefined scheme, thereby reducing the risk of human error. The planned feasibility study is an additional strength, as it will enable us to resolve methodological issues early and identify where modifications are necessary to strengthen trial methodology and operational processes [33].

The planned economic evaluation is important, as financial incentives drive decisions to implement new methods within existing healthcare services [40]. The growing financial burden of cancer care highlights the importance of aligning healthcare resource allocation with clinical benefit across various domains of care [41, 42]. We acknowledge that both registered sickness absence and included cost estimates have inherent limitations, as they may not fully capture changes in disease burden and are often based on approximate or standardised valuations. Consequently, economic evaluation does not directly translate into a clinical decision but does constitute one of several tools to inform it.

A male/female perspective is also highly relevant in this project, as HNC shows substantial sex differences in incidence with a male predominance [1]. This sex difference necessitates the inclusion of both biological and lifestyle factors in the analysis. For example, smoking, alcohol consumption, human papillomavirus (HPV) infections, and healthcare-seeking behaviours may vary between sexes and contribute to variations in disease onset and treatment-related side effects (including oral complications). Identifying potential sex-related differences in functional impairment and the effectiveness of the planned intervention is crucial when evaluating new methods for use in health care.

This study has important clinical implications, and its results will be freely available through open-access journals and plain language summaries for both the public and healthcare professionals. It addresses a growing patient population whose rehabilitation needs are well documented, yet often inadequately met. It also highlights oral health as a critical factor influencing physical, psychological, social, and economic outcomes in HNC survivors. The findings have the potential to inform recommendations for integrating oral care into routine follow-up, thereby promoting equity and structured monitoring of oral health after HNC treatment.

## Supplementary Information

Supplementary Material 1.

## Data Availability

No datasets were generated or analysed during the current study.

## Abbreviations

CRT: Chemoradiotherapy
CT: Computed Tomography
CBCT: Cone-Beam Computed Tomography
DASS-21: Depression Anxiety Stress Scale-21
EORTC-C30: European Organization for Research and Treatment of Cancer Quality of Life Questionnaire-Core 30
EORTC-HN43: European Organization for Research and Treatment of Cancer Quality of Life Questionnaire - Head and Neck Cancer Module
EORTC QLQ-HN35: European Organisation for Research and Treatment of Cancer Quality of Life Questionnaire – Head and Neck Module
GSE: General Self-Efficacy
HNC: Head and neck cancer
HRQoL: Health-related Quality of Life
ITT: Intention-to-treat
MIO: Maximum Interincisal Opening
MiDAS: Micro Data for Analysis of Social Insurance
PROM/s: Patient-reported outcome measure/s
RT: Radiotherapy
RCT: Randomised controlled trial
REDCap: Research Electronic Data Capture
SACT: Systemic anti-cancer therapy
SPIRIT: Standard Protocol Items: Recommendations for Interventional Trials
SPOH: Self-Perceived Oral Health
QALYs: Quality Adjusted Life Years
